# Mimicking and anticipating others’ actions is linked to Social Information Processing

**DOI:** 10.1371/journal.pone.0193743

**Published:** 2018-03-28

**Authors:** Oliver Genschow, Sophie Klomfar, Ine d’Haene, Marcel Brass

**Affiliations:** 1 Social Cognition Center Cologne, University of Cologne, Cologne, Germany; 2 Center for Medical Physics and Biomedical Engineering, Medical University of Vienna, Vienna, Austria; 3 Department of Experimental Psychology, Ghent University, Ghent, Belgium; University of Bologna, ITALY

## Abstract

It is widely known that individuals frequently imitate each other in social situations and that such mimicry fulfills an important social role in the sense that it functions as a social glue. With reference to the anticipated action effect, it has recently been demonstrated that individuals do not only imitate others, but also engage in anticipated action before the observed person starts engaging in that action. Interestingly, both phenomena (i.e., mimicry and anticipated action) rely on tracking others’ social behavior. Therefore, in the present research we investigated whether mimicry and anticipated action are related to social abilities as indicated by measures of social intelligence. The results demonstrate for the first time that mimicry as well as anticipated action is correlated with an important aspect of social intelligence—namely the ability to process social information. Theoretical implications and limitations are discussed.

## Introduction

Individuals imitate a wide range of different behaviors (e.g., [1]) including facial expressions [2], characteristics of language [3–6], emotions (e.g., [7–9]), postures [10], gestures [11], complex action patterns [12] or simple movements [13–17]. In the last decade psychologists investigated the social function of such imitative behavior and demonstrated that imitation acts as a “social glue” (e.g., [18,19]) in the sense that it bonds humans more strongly together (for a more critical view, see [20]). For example, it has been found that individuals have a stronger tendency to automatically imitate pro-social than anti-social gestures [21]. Moreover, imitating others increases pro-social behavior [22], feelings of affiliation [23], or liking for each other [24].

When studying imitation in a naturalistic and social context, researchers investigate mimicry (for a more fine-graded definition, see [25]). Mimicry is usually explained by the so-called perception-behavior link [24,26,27]. This link assumes that mimicry is based on a shared mental representation of perceived and executed action. That is, the observation of an action primes and thus facilitates the execution of a compatible action, because observed and executed actions activate the same motor representation (see also [28–30]). Indeed, the idea of activated representations that are shared between observer and executer has been confirmed extensively in behavioral studies (for a meta-analysis, see [31]), fMRI studies (e.g., [32,33]), motor TMS studies (e.g., [34,35]), and single-cell recordings in monkeys [36] as well as humans [37].

Going one step further, in the last decade, the idea has been put forward that people may not only imitate what others do, but may also anticipate what others might do in the future. For example, predictive coding accounts suggest that predicting others’ behavior is an inherent process that is constantly taking place in human brains in order to prepare one’s own actions (e.g., [38–40]). Wilson and Knoblich [40] propose a so-called emulator that internally simulates others’ action execution. This simulation process then provides immediate information about the ongoing course of the observed action as well as its probable immediate future. Internal modeling allows the observer to rapidly interpret perceptual signals, to react quickly, to disambiguate uncertain situations, and to interpret observed movements that are only partly visible.

Recently, we investigated whether individuals would anticipate others’ action also on the basis of the interpretation of the other person’s nonverbal signals that precede an action and whether such anticipation let people engaging in the anticipated action without the other person ever showing this action [41]. In two studies, participants observed either a video sequence in which a model was wrinkling the nose or a video in which her hair was falling in her face all couple of seconds. While participants were observing the videos, we videotaped them and then coded how often participants engaged in anticipated actions—namely touching the nose when observing the nose wrinkling video or touching the forehead when observing the hair falling video. The results indicated that while watching the nose wrinkling video, participants engaged more often in nose touching than in hair touching and vice versa while watching the hair falling video. In Experiment 2, the model’s desire to engage into the to be anticipated action was manipulated. The results demonstrated that anticipated action is stronger when the observer infers a strong desire to act, compared to when she or he infers a weak desire in the model.

Interestingly, this anticipated action effect resembles some similarities with mimicry. For both phenomena individuals need perceiving, processing and interpreting others’ behavior in order to produce their own behavior. In other words, for both phenomena the tracking of others’ behavior is a crucial precondition. However, an important difference between the two phenomena is how the tracking of another person’s behavior is taking place. While mimicry is triggered by action observation, anticipated action is triggered by the interpretation of contextual events, such as nonverbal cues, that precede an action. Hence, mimicry can be seen as a reactive phenomenon while anticipated action is predictive by nature. Taken together, mimicry and anticipated action differ in important aspects, but show similarities in the sense that both phenomena rely on tracking others’ behavior. This raises the question whether mimicry as well as anticipated action actually correlate with certain personality traits related to the processing of social behavior.

Tracking others’ behavior obviously requires the accurate processing of nonverbal information, which is a key component of social intelligence (e.g., [42]). Social intelligence refers to the ability to interpret other individuals’ behavior in terms of mental states (thoughts, intentions, desires and beliefs), to interact within complex social groups and in close relationships, to empathize with others’ states of mind, and to predict how others will feel, think and behave [43]. Silvera and colleagues [42] distinguish three different subfactors of social intelligence including Social Skills, Social Awareness, and Social Information Processing. Social Skills relates to one’s ease at getting along with others in social situations and Social Awareness relates to one’s general awareness of one’s own role in social interactions [44]. Social Information Processing involves the understanding of both implicit and explicit verbal and nonverbal messages [45], as well as the “ability to predict someone’s feelings and behavior” ([46] p. 262). Based on this definition and given that mimicry and anticipated action rely on tracking others’ social behavior, it is reasonable to assume that at least the “Social Information Processing” subscale of social intelligence is related to both, mimicry and anticipated action.

### Present research

The primary goal of the current study was to test whether anticipated action is related to subfactors of social intelligence. In addition, we aimed at testing the degree to which mimicry is related to anticipated action and social intelligence in an exploratory fashion. Thus, we chose a design in which we were able to assess anticipated action as well as mimicry.

## Method

### Ethics statement

The study was conducted in accordance with the ethical standards of the 1964 Declaration of Helsinki and approved by the rules of the Institutional Review Board from the Faculty of Psychology and Educational Science of Ghent University (Belgium). All participants provided informed consent at the beginning of the experiment and were informed that participation was voluntary and that all answers were processed and stored anonymously.

### Participants

81 students (70 female) of Ghent University (Belgium) with an age ranging from 17 to 36 (*M* = 20.30; *SD* = 3.58) participated in return for partial course credit into the study.

### Procedure

The experiment was the first experiment in a row of other experiments. After being welcomed, participants were seated in separate cubicles, signed an informed consent and then ran through the experiment. Instructions and stimuli were presented on a computer screen. Specifically, participants were told that they would observe three video clips in which a woman reads a story. We told participants that they should listen carefully to the story, as questions to the text have to be answered at the end of the experiment. Participants then watched the three video clips (total duration 30 min) of a female model reading a story from the Dutch children book titled “Pluk van de Petteflet” [47]. The story was about a boy driving an ambulance. We made sure that in none of the videos the story’s content was related to nose wrinkling, itching or touching. The female model who read the story wore a black t-shirt and no make-up. The first video served as baseline measure in which the model was reading the story in a neutral fashion. Within the second video we assessed anticipated action. That is the model engaged every 30 seconds in nose wrinkling actions while reading. Finally, the third video was established to measure participants’ mimicking behavior. Therefore, while reading, the model engaged in nose touching actions every 30 seconds. While participants observed the three video parts we videotaped them. Afterwards, a coder blind to the conditions coded how often each participant touched his or her nose in each condition. In line with previous research (e.g., [48]), we computed for each observed video an action score by dividing the amount of executed nose touching actions by the duration of the respective video. Each participant, therefore, had three scores (i.e., one score for each condition) that reflected the frequency per minute of executed nose touching actions.

After participants observed the video, we assessed the Dutch version [49] of the Tromsø Social Intelligence Scale (TSIS; [42]). The TSIS consists of 21 statements. For each statement, participants rated the degree to which it described them on a scale from 1 to 7 (1 = *describes me extremely poorly*; 7 = *describes me extremely well*). The TSIS differentiates between three subscales: Social Awareness (e.g., I have often hurt others without realizing it), Social Skills (e.g., I am good at entering new situations and meeting people for the first time), and Social Information Processing (e.g., I can often understand what others are trying to accomplish without the need for them to say anything.). To prepare data for analysis we computed composite scores for the subscales Social Awareness (Cronbach’s α = .74), Social Skills (Cronbach’s α = .58) and Social Information Processing (Cronbach’s α = 75).

## Results

### Investigation of anticipated action and mimicry

**Fig 1:**
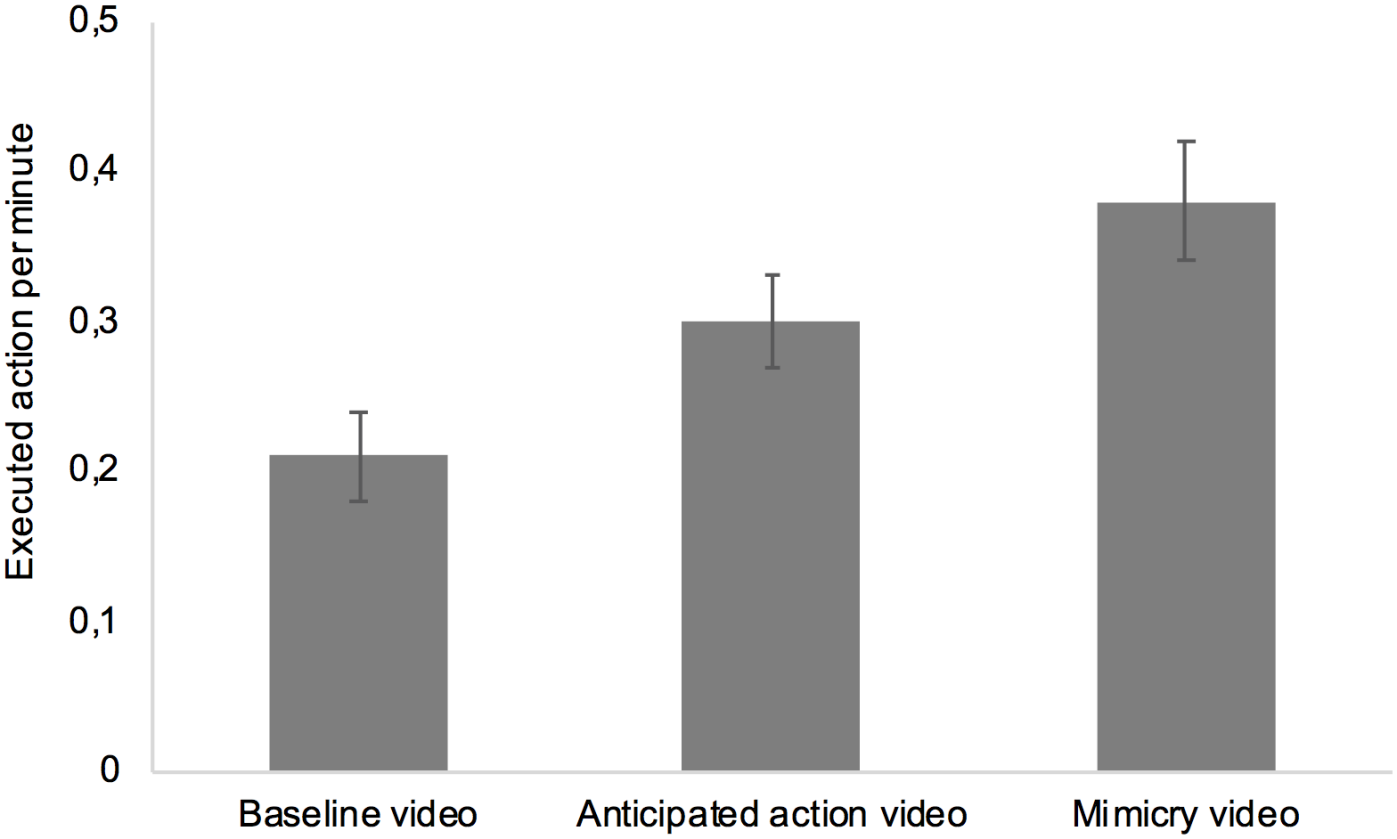
Amount of executed nose touching actions per minute as a function of observed video. Error bars represent standard errors.

In a first step, we tested the experimental hypothesis that the nose wrinkling video and the nose touching video increase participants’ number of nose touching actions by running a one-factorial (video type: baseline vs. nose wrinkling vs. nose touching) ANOVA. The analysis revealed a significant effect of video type on participants’ nose touching actions, *F* (2, 80) = 10.86, *p* < 0.001, η_p_^2^ = .12 (see Fig 1). Consistent with the predictions, planned comparisons revealed that participants engaged in more nose touching per minute when observing the model engaging in nose wrinkling (*M* = 0.30, *SD* = 0.28) than when observing the model reading the story in a neutral way (*M* = 0.21, *SD* = 0.26), *t*(80) = 2.86, *p* = .005, *dz* = .33, providing evidence for anticipated action. In terms of mimicry, we found that participants engaged in more nose touching per minute when observing the model touching the nose (*M* = 0.38, *SD* = 0.34) than when observing the model reading the story in a neutral fashion (*M* = 0.21, *SD* = 0.26), *t*(80) = 4.19, *p* < .001, *dz* = .48, indicating a mimicry effect.

In a second step, we tested whether there are differences between the amount of anticipated action and amount of mimicking behavior. That is, we ran a one-factorial (video type: nose wrinkling vs. nose touching) ANOVA and entered the baseline condition as covariate in order to control for natural occurring nose touching actions. The analysis revealed a main effect for video type, *F* (1, 79) = 4.28, *p* = .042, η_p_^2^ = .05, indicating that participants engaged in stronger mimicking behavior than anticipated action. To further test this interpretation, we calculated a baseline corrected anticipated action effect by subtracting the baseline condition from the anticipated action condition. Similarly, we calculated a baseline corrected mimicry effect by subtracting the baseline condition from the mimicry condition. In line with the ANOVA, a t-test for dependent samples indicates that participants showed a stronger mimicry effect (*M* = .16, *SD* = .35) than anticipated action effect (*M* = 0.09, *SD* = 0.27), *t*(80) = 2.16, *p* = .033, *dz* = .22.

In a third step, we tested whether the baseline corrected anticipated action effect and the baseline corrected mimicry effect are correlated. The analysis yielded a positive correlation, *r* = .50, *p* < .001, indicating that the more individuals anticipate another person’s action, the more they mimic this person.

### Relation of social intelligence with anticipated action and mimicry

**Fig 2:**
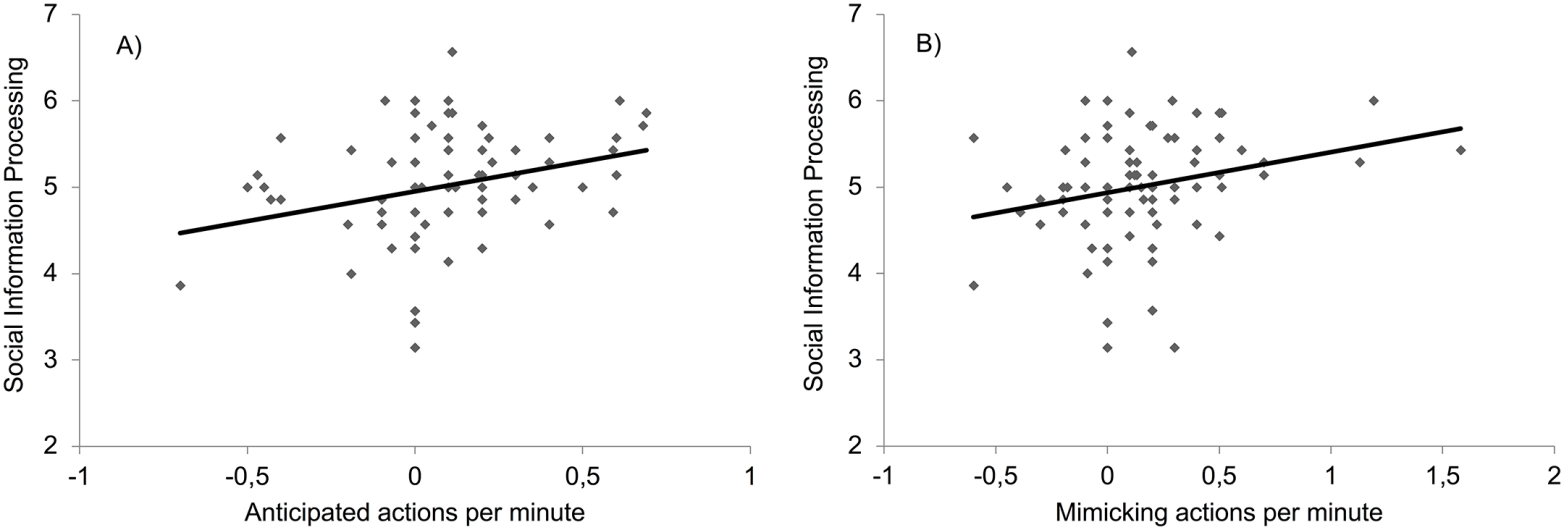
Correlation between Social Information Processing and the baseline corrected anticipated action effect (A) and the baseline corrected mimicry effect (B).

In order to test whether anticipated action and mimicry is related to social intelligence we ran correlation analyses with all assessed subscales of social intelligence as well as the baseline corrected anticipated action effect and the baseline corrected mimicry effect (see Table 1 for an overview of the correlational matrix). As can be seen in Fig 2, Anticipated action (*r* = .29, *p* = .009), as well as mimicry (*r* = .25, *p* = .023) significantly correlated with Social Information Processing. No other subscale of social intelligence correlated with anticipated action or with mimicry (*r* < .12, *p* > .29).

**Table 1:** Intercorrelations between anticipated action, mimicry and subscales of social intelligence.

	1.	2.	3.	4.	5.
1. Anticipated action	-	.50**	.29**	.12	-.05
2. Mimicry		-	.25*	.01	-.01
3. SI			-	.20	-.21
4. SS				-	.25*
5. SA					-

* *p* < .05.

**p < .01.

Note. SI = Social Information Processing; SS = Social Skills; SA = Social Awareness

Although Social Information Processing correlated with anticipated action and mimicry, one could argue that individuals who are better in processing social information are generally more active in terms of their motor behavior. In order to test this alternative explanation, we ran additional correlations between all subscales of the social intelligence scale and the amount of nose touching actions per minute in the baseline condition. The results indicate that none of the subscales is significantly correlated with the amount of nose touching actions (*r* < .12, *p* > .30) suggesting that social information processing as part of social intelligence is linked to mimicry and anticipated action, but not to motor behavior in general.

### Additional control analyses

It needs to be mentioned that in our experiment, there were two groups of participants. Within one group of participants the baseline video was only 5 minutes long and within the other group of participants the baseline video was 10 minutes long. In order to test whether the difference in the length of the baseline videos had an effect on mimicry and anticipated action we ran a 3 (video: baseline vs. anticipation vs. imitation) x 2 (group: 5 minutes baseline vs. 10 minutes baseline) mixed ANOVA with the first factor as within-subject and the second factor as between-subject factor. The interaction between video and group was not significant, *F* (1, 79) = 0.24, *p* = 0.78, indicating that the different groups had no influence on mimicry and imitation. This interpretation is supported by a t-test for independent samples finding no significant difference between the amount of nose touching actions per minute in the 5 minutes lasting baseline video (*M* = 0.22, *SD* = 0.26) as compared to the 10 minutes lasting baseline video (*M* = 0.20, *SD* = 0.26), *t*(79) = 0.39, *p* = .70.

## Discussion

Although mimicry and anticipated action share similar processes, they differ in important aspects. While for both phenomena the tracking of others’ behavior is a crucial precondition, the process is triggered in different ways. While mimicry is triggered by action observation, anticipated action is triggered by the observation of nonverbal expressions preceding an action. The present study investigated for the first time whether the two different phenomena are linked to certain personality traits related to the processing of social behavior. To this end, we investigated whether mimicry and anticipated action are related to social intelligence. The results demonstrate that Social Information Processing as part of social intelligence is positively linked to anticipated action as well as to mimicry. No other subfactor of social intelligence was significantly correlated with mimicry or anticipated action.

To the best of our knowledge, this is the first study that demonstrates an association of Social Information Processing as part of social intelligence with mimicry and anticipated action. The finding that Social Information Processing positively correlates with mimicry as well as anticipated action is in line with past research demonstrating the moderating influence of social factors within mimicking behavior. For example, it has been shown that empathy (for a review, see [50]), perspective taking [15,24], pro-social orientation [51–54], interdependent self-construal [48] or field-dependence [55] positively correlate with mimicry. Within this line of research, our findings demonstrate the strong social component of mimicry. Indeed, past research suggests that mimicking others has positive social consequences linking persons more strongly together (for an overview see [19]). Given that anticipated action shares processes with mimicry and both phenomena are moderated by Social Information Processing, it might well be that anticipated action has similar positive social consequences as mimicry. Future research may further investigate this interpretation. Nevertheless, it is important to note that there are also studies indicating that mimicry is not as strongly correlated to personality factors, such as empathy, as it has been previously assumed (e.g., [25]). Therefore, future research should aim at replicating our findings and test whether social information processing correlates also with other forms of imitation.

Our primary goal of the present study was to investigate the relation between social intelligence and anticipated action. Beside the positive correlation, in secondary analyses, we also investigated the relation between anticipated action and mimicry. The results indicate that mimicry and anticipated action are positively correlated with each other and, thus, offer interesting theoretical implications. Past research demonstrated that mimicry is based on the direct simulation of others’ behavior [26,29,30]. Thereby, observing another person’s action activates motor representation similar to those of the actor (e.g., [56]) facilitating the execution of compatible actions [13]. The finding that anticipative processes are linked to mimicry allows the speculation that the process of mimicking others might already be triggered before the other person has started acting. That is, when observers are able to anticipate what the other person wants do in the future, the process of mimicking this action might already be initiated. However, our data also suggest that mimicry cannot be fully explained by anticipative processes, because the mimicry effect is more strongly pronounced than the anticipation effect. Therefore, we suggest that mimicry can be initiated by correctly anticipating what the other person may do in the future. However, anticipating others’ action itself may not be a sufficient precondition for mimicry to occur, but has the potential to increase mimicking tendencies.

Despite this theoretical implication, our methodological approach also calls for a more careful discussion. That is, we have to acknowledge the possibility of order effects in our study, because the order of anticipated action and mimicry was not counterbalanced. Although we regard it as very unlikely, it could still be that the anticipated action block, which preceded the mimicry block, primed the execution of nose touching in such a way that the mimicking of nose touching was already pre-activated. Therefore, the assessment of mimicry might be noisier than we have aimed for. This would indicate that the correlation between mimicry and anticipated action, but also the correlation between mimicry and Social Information Processing should be interpreted with caution. Thus, we consider the relation of mimicry with anticipated action as well as with Social Information Processing as preliminary evidence that needs to be replicated in better controlled designs. It is important to note, however, that the main goal of the present research was to investigate the relationship of anticipated action and subfactors of social intelligence and that the correlation between anticipated action and Social Information Processing is not affected by the above-mentioned potential shortcoming and can still be interpreted.

Yet another concern regarding our study might be that our sample size was not large enough. We expected a medium too large effect for which our sample was sufficient. However, it might be that we overestimated the expected effect size. Thus, our findings should be taken as initial evidence and future research should aim at replicating our findings with larger samples.

Relatedly, we have to acknowledge that our sample was rather gender-biased with more women than men participating in our study. Thus, an interesting question is whether our results could be replicated within male participants. Previous studies applying similar tasks within similar populations did not find any gender differences [25,41]. However, Meijs et al. [49] found in the Tromsø Social Intelligence Scale gender differences with men scoring lower than women. This raises the question whether the correlation between social intelligence and anticipated action as well as mimicry differs between men and women. While our data do not allow answering this question, future research should aim at testing this question more rigorously.

Finally, an interesting question concerns the process underlying anticipated action. We assume that observing nose wrinkling actions activates the anticipated action—that is nose touching. In line with this idea we found that participants touched their nose more often when observing another person’s wrinkling her nose while reading a story than when observing a person reading in a neutral fashion. Past research indicates that anticipated action is at least partially based on the inferred desire to act [41]. However, even if the model expresses no desire to act, individuals engage in anticipated action. Therefore, it might well be that in addition to an inferred desire, individuals simulate nose wrinkling, which then facilitates nose touching. As we did not measure such simulation process in the present research we were not able to test this assumption. However, future research may aim at testing this relation more directly. Moreover, future research should test to which degree social information processing is related to inferring individuals’ desire to act or to simulating the triggering event.

### Summary

Mimicry and anticipated action rely on the tracking of others’ behavior. In line with this notion, in the present study we investigated whether the two phenomena are linked to personality traits that are related to the processing of social behavior and found that mimicry and anticipated action correlate with the information processing aspect of social intelligence. While the correlation between anticipated action and Social Information Processing has interesting implications the correlation between mimicry and Social Information Processing should be taken with caution due to possible order effects.
